# Comment on Wijnia, J.W. A Clinician’s View of Wernicke-Korsakoff Syndrome. *J. Clin. Med.* 2022, *11*, 6755

**DOI:** 10.3390/jcm12196393

**Published:** 2023-10-07

**Authors:** Michele Manigrasso, Nunzio Velotti, Giovanni Domenico De Palma, Mario Musella

**Affiliations:** 1 Department of Clinical Medicine and Surgery, “Federico II” University of Naples, Via Sergio Pansini 5, 80131 Naples, Italy; giovanni.depalma@unina.it; 2 Department of Advanced Biomedical Sciences, “Federico II” University of Naples, Via Sergio Pansini 5, 80131 Naples, Italy; nunzio.velotti@gmail.com (N.V.); mario.musella@unina.it (M.M.)

We have read with great interest the article by Wijnia [1], who analysed the pathophysiology and the clinical aspects of Wernicke–Korsakoff Syndrome (WKS). Specifically, the authors analysed the principal causes of this disease, distinguishing between alcoholic and non-alcoholic Wernicke encephalopathy. Among the non-alcoholic causes of the Wernicke encephalopathy, the authors cited thiamine deficiency after bariatric surgery.

Obesity has to be considered as a risk factor, per se; an increased caloric intake induces an increased load on the metabolic pathways, in particular glucose metabolism, and such metabolism requires micronutrients such as thiamine as enzyme co-factors. Therefore, there is now increasing evidence that the obese population may nutritionally depleted of essential micronutrients. Thiamine deficiency has been reported to be in the region of 16–47% among patients who are candidates for bariatric surgery [2,3].

A previous review by Oudman et al. [4] reported that the major causes of non-alcoholic WKS in adult patients are vomiting and extreme weight loss. In this setting, bariatric surgery plays a fundamental role, being associated with nutritional impoverishment and thiamine deficiency.

Several studies have demonstrated the onset of WKS after bariatric surgery [5,6,7,8,9].

In the NEUROBAR study [5], which analysed 38 patients with neurological complications after bariatric surgery, the majority of them complained of vomiting after surgery (53%) and suffered with postoperative complications (53%). Furthermore, thiamine deficiency was very common, and occurred in 74% of patients, both after sleeve gastrectomy and gastric bypass.

In a systematic review on restrictive procedures, Milone et al. [6] confirmed that postoperative vomiting could be considered a major cause of thiamine deficiency, and consequently, the onset of WKS. In addition, the authors suggested that early thiamine supplementation could improve clinical conditions, thereby preventing permanent deficiencies.

Similarly, several case reports have demonstrated the possibility of a late onset of thiamine deficiency in patients who undergo bariatric surgery, both after restrictive and malabsorptive interventions [7,8,9].

First, Velasco et al. [7] reported the onset of neurological symptoms seven years after vertical banding gastroplasty, and Milone et al. [8] reported a case of Wernicke encephalopathy after sleeve gastrectomy in a patient with postoperative vomiting; finally, Negri et al. [9] described a case of walking disorder in a patient who underwent biliopancreatic diversion six years earlier. 

As stated by Albaugh et al. [10], patients with thiamine deficiency tend to be females with a higher body mass index, while male sex and greater height were both associated with a higher thiamine concentration. From this point of view, the authors concluded that routine thiamine measurement, either preoperatively or at the time of surgery, is warranted, given the potential catastrophic complications associated with acute or chronic deficiency. The authors also stated the thiamine concentration was normally distributed, with a mean of 144 nM, and 3.5% of patients had thiamine concentrations below the lower limit of normal of <70 nM. In this context, the dosing regimen in the deficiency setting should be at least of 120–150 nM per day. 

Despite this, a recent survey on the screening and treatment of thiamine deficiency in a sample of multidisciplinary bariatric surgery clinical teams revealed that only 36% reported routine pre-operative screening for all bariatric patients, and 39% for those post operatively. In 6% of cases, thiamine levels were only assessed in the presence of conditions such as vomiting, poor dietary intake, poor nutrition, or paraesthesia, or if clinically indicated [11].

On the basis of the most recent literature, recent guidelines have stated the importance of thiamine therapy after bariatric surgery, especially after malabsorptive procedures [12], even if the existing literature also suggests the possibility of thiamine deficiency in patients who have undergone restrictive bariatric procedures. 

Thus, considering the incidence of this disease, especially in young patients, we think that the impact of bariatric surgery should be more extensively discussed when analysing the aetiology of non-alcoholic WKS.

Similarly, from a clinical point of view, requests for deeper analyses of the past medical histories of patients with Wernicke encephalopathy must be made; we must recognize thiamine deficiency more rapidly, and start immediate thiamine therapy to avoid permanent neurological damage.

## References

[1] Wijnia J.W. (2022). A Clinician’s View of Wernicke-Korsakoff Syndrome. J. Clin. Med..

[2] Kerns J.C., Arundel C., Chawla L.S. (2015). Thiamin deficiency in people with obesity. Adv. Nutr..

[3] Maguire D., Talwar D., Shiels P.G., McMillan D. (2018). The role of thiamine dependent enzymes in obesity and obesity related chronic disease states: A systematic review. Clin. Nutr. ESPEN.

[4] Oudman E., Wijnia J.W., Oey M.J., van Dam M., Postma A. (2021). Wernicke-Korsakoff syndrome despite no alcohol abuse: A summary of systematic reports. J. Neurol. Sci..

[5] Alligier M., Borel A.L., Savey V., Rives-Lange C., Brindisi M.C., Piguel X., Nocca D., Monsaingeon-Henry M., Montastier E., Beliard S. (2020). A series of severe neurologic complications after bariatric surgery in France: The NEUROBAR Study. Surg. Obes. Relat. Dis..

[6] Milone M., Di Minno M.N., Lupoli R., Maietta P., Bianco P., Pisapia A., Gaudioso D., Taffuri C., Milone F., Musella M. (2014). Wernicke encephalopathy in subjects undergoing restrictive weight loss surgery: A systematic review of literature data. Eur. Eat. Disord. Rev..

[7] Velasco M.V., Casanova I., Sanchez-Pernaute A., Pérez-Aguirre E., Torres A., Puerta J., Cabrerizo L., Rubio M.A. (2009). Unusual late-onset Wernicke’s encephalopathy following vertical banded gastroplasty. Obes. Surg..

[8] Scarano V., Milone M., Di Minno M.N., Panariello G., Bertogliatti S., Terracciano M., Orlando V., Florio C., Leongito M., Lupoli R. (2012). Late micronutrient deficiency and neurological dysfunction after laparoscopic sleeve gastrectomy: A case report. Eur. J. Clin. Nutr..

[9] Negri M., Macerola N., Mancarella F.A., Bruno C., De Leva F., D’Argento F., Pontecorvi A., Modoni A., Tartaglione L., Di Leo M. (2019). A Late Onset of Wernicke-Korsakoff Encephalopathy after Biliopancreatic Diversion: A Case Report. Obes. Surg..

[10] Albaugh V.L., Williams D.B., Aher C.V., Spann M.D., English W.J. (2021). Prevalence of thiamine deficiency is significant in patients undergoing primary bariatric surgery. Surg. Obes. Relat. Dis..

[11] Zarshenas N., Tapsell L.C., Batterham M., Neale E.P., Talbot M.L. (2021). Screening and treatment of thiamine deficiency in a sample of multidisciplinary bariatric surgery clinical teams. Obes. Surg..

[12] Mechanick J.I., Apovian C., Brethauer S., Garvey W.T., Joffe A.M., Kim J., Kushner R.F., Lindquist R., Pessah-Pollack R., Seger J. (2020). Clinical practice guidelines for the perioperative nutrition, metabolic, and nonsurgical support of patients undergoing bariatric procedures—2019 update: Cosponsored by American Association of Clinical Endocrinologists/American College of Endocrinology, The Obesity Society, American Society for Metabolic & Bariatric Surgery, Obesity Medicine Association, and American Society of Anesthesiologists. Surg. Obes. Relat. Dis..

